# Engineering two-dimensional superconductivity and Rashba spin–orbit coupling in LaAlO_3_/SrTiO_3_ quantum wells by selective orbital occupancy

**DOI:** 10.1038/ncomms7028

**Published:** 2015-01-13

**Authors:** Gervasi Herranz, Gyanendra Singh, Nicolas Bergeal, Alexis Jouan, Jérôme Lesueur, Jaume Gázquez, María Varela, Mateusz Scigaj, Nico Dix, Florencio Sánchez, Josep Fontcuberta

**Affiliations:** 1 Institut de Ciència de Materials de Barcelona (ICMAB-CSIC), Campus de la UAB, 08193 Bellaterra, Catalonia, Spain; 2 LPEM-UMR8213/CNRS-ESPCI ParisTech-UPMC, PSL University, 10 rue Vauquelin, 75005 Paris, France; 3 Materials Science & Technology Division, Oak Ridge National Laboratory, Oak Ridge, Tennessee 37831, USA; 4 GFMC, Department de Fisica Aplicada III & Instituto Pluridisciplinar, Universidad Complutense de Madrid, 28040 Madrid, Spain; 5 Departament de Física, Universitat Autònoma de Barcelona, E-08193 Bellaterra, Barcelona, Catalonia, Spain

## Abstract

The discovery of two-dimensional electron gases (2DEGs) at oxide interfaces—involving electrons in narrow *d*-bands—has broken new ground, enabling the access to correlated states that are unreachable in conventional semiconductors based on *s*- and *p*- electrons. There is a growing consensus that emerging properties at these novel quantum wells—such as 2D superconductivity and magnetism—are intimately connected to specific orbital symmetries in the 2DEG sub-band structure. Here we show that crystal orientation allows selective orbital occupancy, disclosing unprecedented ways to tailor the 2DEG properties. By carrying out electrostatic gating experiments in LaAlO_3_/SrTiO_3_ wells of different crystal orientations, we show that the spatial extension and anisotropy of the 2D superconductivity and the Rashba spin–orbit field can be largely modulated by controlling the 2DEG sub-band filling. Such an orientational tuning expands the possibilities for electronic engineering of 2DEGs at LaAlO_3_/SrTiO_3_ interfaces.

## Abstract (web summary)

Two-dimensional electron gases at oxide interfaces induce exotic behaviours. By studying samples with different crystal orientation, Herranz *et al.* show that the extension and anisotropy of the oxide quantum well properties can be controlled through selective sub-band filling via orientational tuning.

## Graphical abstract

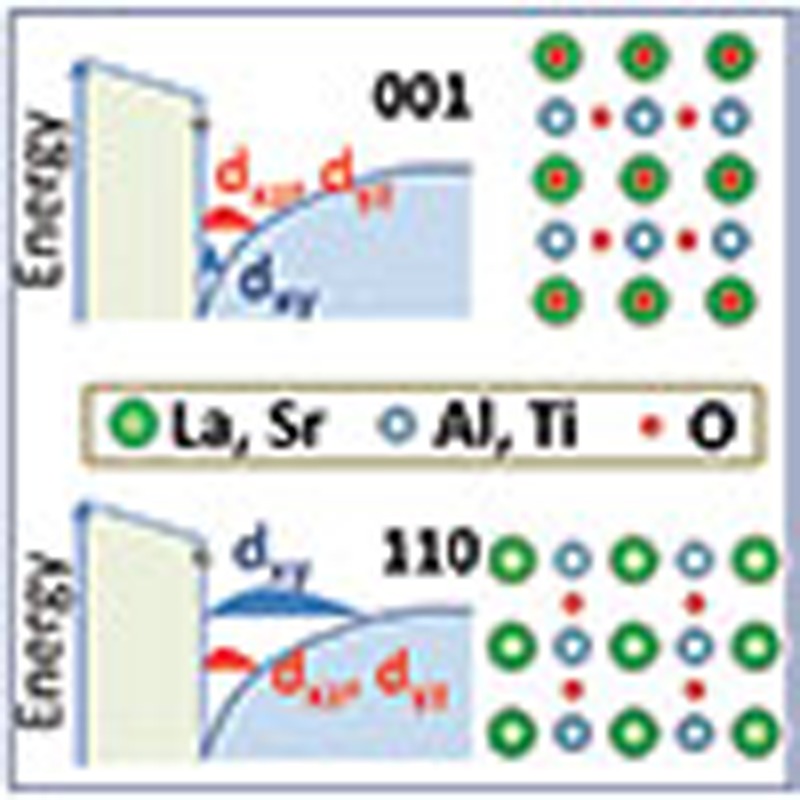

The confinement of electron orbitals over small scales provides a pathway to tailor the electronic properties of a quantum system. Restricting the motion of electrons within planes of different crystal orientation affords additional routes to reorganize the electronic band structure. Such strategies have been used to engineer the electronic and optical properties of II–VI and III–V semiconductor quantum wells, where *s*- and *p*- orbitals are involved^1^,^2^,^3^. Yet, two-dimensional (2D) electron gases (2DEGs), comprising *d*-electrons instead of *s* or *p*, have come into the limelight over about the last 10 years^4^,^5^,^6^,^7^,^8^,^9^,^10^,^11^, opening novel perspectives that are inaccessible for more traditional materials. In particular, the narrow bandwidth of *d*-states in transition metal oxide quantum wells promote correlated states—for example, magnetism and superconductivity—that are unseen in conventional semiconductors. Interestingly, the extreme confinement, over just a few unit cells, enables full electrostatic control of correlated 2DEG states, allowing access to new physics and paving the way to new device concepts. Particularly, these 2D electron systems have been found to be superconductive^7^,^12^,^13^,^14^, with 2D superconductivity largely modulated by electric gates^15^,^16^. The 2D character of the superconductivity has led to phenomena not observed in the 3D regime, such as magnetic enhancement of superconductivity^17^, violation of the paramagnetic Pauli limit for the upper critical fields^18^, quantum phase transitions^19^ or multiple quantum criticality^20^. The intricacy of all these complex phases and the evidence of the role of electron correlations have often prompted the use of the concept of electron liquids to designate these electron systems^21^.

The interface between LaAlO_3_ and SrTiO_3_ is the oxide quantum well par excellence. Initially, the research on LaAlO_3_/SrTiO_3_ quantum wells was restricted to the (001)-plane of the perovskite unit cell^4^,^14^,^15^. Remarkably, recent investigations have uncovered that interface conductivity also appears along other directions, such as (110) (refs. 22, 23) and (111) (ref. 22). The selective confinement of electrons within planes of different crystal orientation expands vigorously the possibility of fine-tuning the 2DEG sub-band hierarchy and, thereof, the physical properties. Along this line, we have recently demonstrated that crystal symmetry is an extra degree of freedom to realize different 2DEG band reconstructions at the LaAlO_3_/SrTiO_3_ interface, by imposing distinctive orbital hierarchies on (001)- and (110)-oriented quantum wells and enabling the selective occupancy of states of different symmetry^24^. More specifically, we have uncovered that the degeneracy within the *t*_2g_ sub-band—which forms the backbone of the 2DEG structure in LaAlO_3_/SrTiO_3_ wells—is broken in reversed ways depending on the crystal orientation: for (001)-oriented 2DEGs the *d*_*xy*_ orbitals have the lowest energy, while along (110) the bottommost levels have instead a *d*_*xz*_*/d*_*yz*_ character^24^. Recent experiments on uncapped (110) SrTiO_3_ surfaces also found the same hierarchy^25^. This orbital reconfiguration provides an excellent playground to test the link between orbital symmetry and complex correlated states, provided that we understand exactly the implications that such 2DEG band engineering has for the physical properties of the quantum wells.

In this work, we present evidence that the selection of the orbital symmetries in the 2DEG sub-band structure triggers some nontrivial and extensive modifications of the electronic properties of quantum wells at the LaAlO_3_/SrTiO_3_ interface. First, we demonstrate that the orbital reconfiguration implies a modulation of 2DEG spatial extension and, as a result, the anisotropy of the 2D superconductivity is largely affected by crystal orientation. Second, we show that the effects of sub-band engineering are influential on the spin–orbit coupling and the concomitant Rashba effect, opening new pathways to tune the spin–dependent transport in LaAlO_3_/SrTiO_3_ quantum wells. These findings open fresh perspectives to understand the fundamental connection between orbital symmetry and the electronic phases at LaAlO_3_/SrTiO_3_ interfaces.

## Results

### Structural characterization

**Figure 1:**
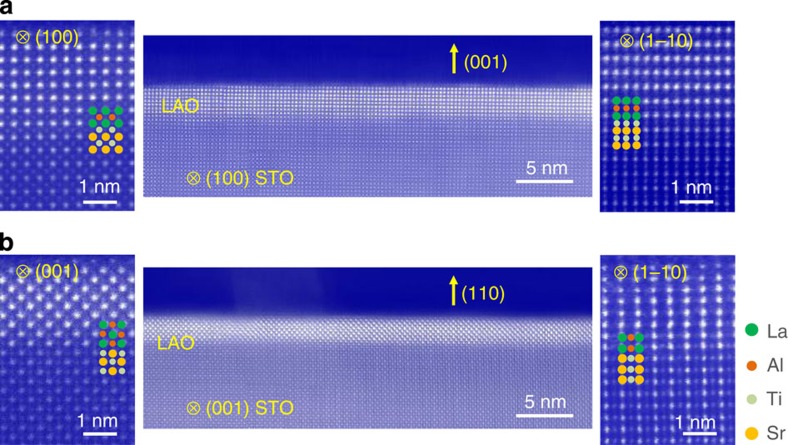
Atomic-resolution STEM characterization. (**a**) HAADF-STEM images of the LaAlO_3_/SrTiO_3_ (001) interface. The left and right panels are magnified views of the interface observed from [100] and [1–10] zone axes, respectively. (**b**) HAADF-STEM images of the LaAlO_3_/SrTiO_3_ (110) interface. Left and right panels are magnified views of the interface observed from [001] and [1–10] directions, respectively. Both LaAlO_3_ layers are continuous within the analysed region (of the order of 1 μm). The images in the central panels **a** and **b** have been Fourier filtered to reduce background noise. The positions of La and Sr are indicated by green and orange circles, whereas Al and Ti are shown in red and light green. Note that for both orientations the interfaces are atomically flat and that the (110) interface does not show any local (100) microfacet.

The samples analysed here were obtained by pulsed laser deposition of LaAlO_3_ thin films on TiO_2_-terminated (001)-SrTiO_3_ substrates (LaAlO_3_ thickness *t*=10 monolayers (MLs), corresponding to *t*~3.8 nm) as well as on thermally treated (110)-oriented SrTiO_3_ substrates (*t*=7–14 MLs, *t*~1.9–3.8 nm), see details in Methods and (refs 22, 26, 27). We carried out cross-sectional scanning transmission electron microscopy (STEM) in the high-angle annular dark field (HAADF) imaging mode, in which, to a good approximation, the intensity of an atomic column is proportional to the square of the atomic number (*Z*), so elements can be deduced by tracking column intensities^28^. Brighter atomic columns correspond to the heavier elements, La and Sr, whereas fainter columns correspond to Ti and Al. Atomic-scale structural characterization shows a coherent and epitaxial growth of both heterostructures and atomically flat interfaces—Fig. 1a,b for (001) and (110), respectively— Besides, regarding the (110)-oriented sample, along the [001] zone axis the (110) ionic stacking across the interface can be readily appreciated, see Fig. 1b. Therefore, in spite of the higher surface energies of (110)-planes with respect to (001), the STEM-HAADF study rules out altogether the formation of (100) microfacets at the (110)-interface^23^,^29^,^30^.

### Spatial extension and anisotropy of 2D superconductivity

**Figure 2:**
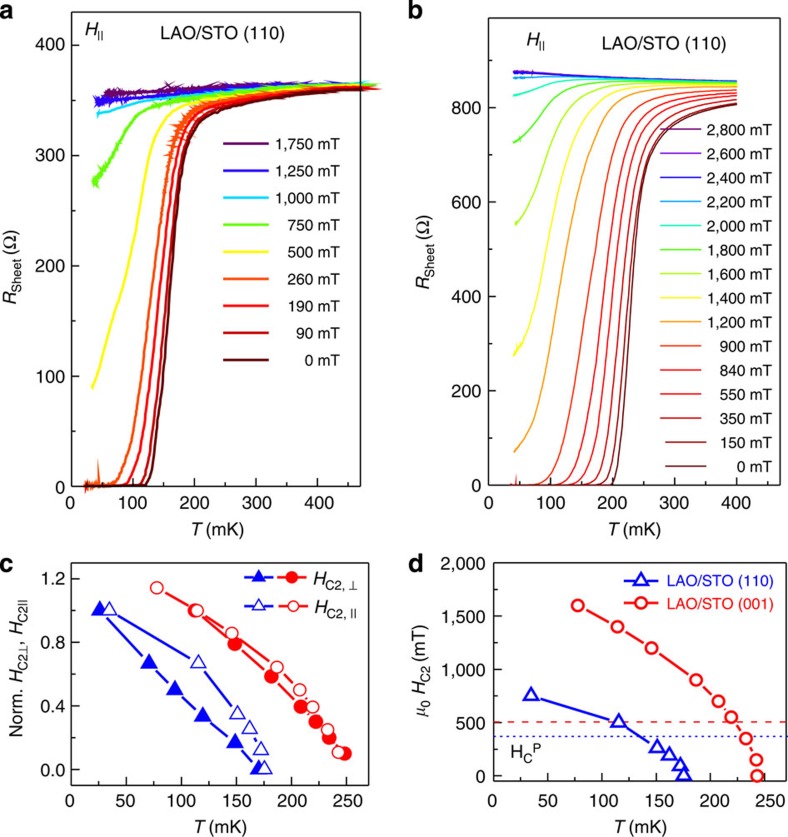
Anisotropy of the 2D superconductivity at the (001) and (110) interfaces. Sheet resistance of (**a**) the (110)-interface with *t*=14 MLs and (**b**) the (001)-interface with *t*=10 MLs, under magnetic fields applied parallel to the interface. The field values are indicated in the panels. Panel (**c**) shows the temperature dependence of out-of-plane *μ*_0_*H*_c2,⊥_ and in-plane *μ*_0_*H*_c2,‖_ critical fields of (001)—red circles—and (110)—blue triangles—interfaces, corroborating the 2D character of the superconductivity for both orientations. (**d**) The upper critical fields are displayed as a function of the temperature for both the orientations (field in-plane). The dotted and dashed straight lines indicate the Pauli-limited critical fields 
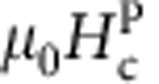
. The observation of higher critical fields for the (001) interface is consistent with the larger anisotropy of the 2DEG superconductivity and stronger spatial confinement for (001).

We discuss first the implications of band reconstruction on the 2DEG superconductivity. In line with previous reports on (001) (refs 14, 15, 19), we show that the (110)-interface is also superconductive and has a 2D character. Yet, we uncover that the anisotropy of the 2D superconductive state is considerably larger for (001) than for (110). Such a conclusion is readily apparent from the sheet resistance curves measured under the magnetic fields applied in-plane (Fig. 2a,b). It is known that as the 2D limit is approached, increasingly higher in-plane fields are required to suppress the superconductivity, since vortex entry is impeded by the low dimensionality^13^. Therefore, higher in-plane critical fields imply stronger anisotropy. Inspection of Fig. 2a,b shows that the (001) interface requires much higher in-plane fields (*μ*_0_*H*_c2,‖_≈2,200 mT) than the (110) interface (*μ*_0_*H*_c2,‖_≈1,000 mT) to induce the transition to the normal state. We conclude, thus, that the 2D anisotropy is larger for (001) than for (110), anticipating a smaller spatial extension of the quantum well along (001).

For a quantitative estimation of both the superconductive layer thickness *d* and the in-plane superconductive coherence length *ξ*, we carried out an analysis based on the Landau–Ginzburg formalism^31^. For that purpose, the out-of-plane *μ*_0_*H*_c2,⊥_ and in-plane *μ*_0_*H*_c2,‖_ critical fields were determined by defining quantitative criteria for the field-induced transitions. Thus, a drop resistance of 90% from the normal resistance state at *T*=400 mK was established to ascertain the evolution of the transition temperature *T*_C_. We consider first the (110) sample with LaAlO_3_ thickness *t*=14 MLs. The out-of-plane critical field, extrapolated to *T*=0 K, was *μ*_0_*H*_c2,⊥_≈160 mT (Supplementary Fig. 1), leading to an in-plane coherence length 
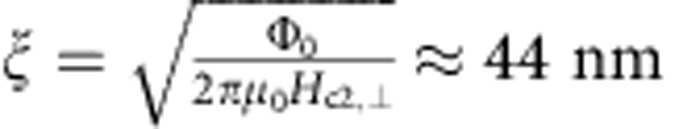
 (*Φ*_0_ is the flux quantum)^31^. In addition, from the in-plane critical field *μ*_0_*H*_c2,‖_≈1,000 mT we could estimate the superconductive thickness 
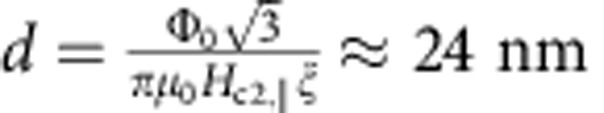
. Since the coherence length is well above the superconductive thickness (*d*<*ξ*), the superconductivity is shown to be 2D. Applying the same protocol analysis to the other (110)-interfaces in this study, with thickness in the range *t*=7–10 MLs (Supplementary Fig. 2), we find that always the in-plane coherence length (*ξ*≈40–75 nm) is significantly larger than the superconductive thickness (*d*≈24–30 nm), thus confirming the 2D character of the superconductivity at the (110) interface. This is also corroborated by the analysis of the temperature dependence of the resistance, showing that the transition to the superconductivity at (110) interfaces belongs to the Berezinskii–Kosterlitz–Thouless (BKT) universality class^32^,^33^,^34^ (Supplementary Fig. 3). In addition, the out-of-plane and in-plane critical fields follow the temperature dependence expected for 2D superconductors (Fig. 2c), that is, 
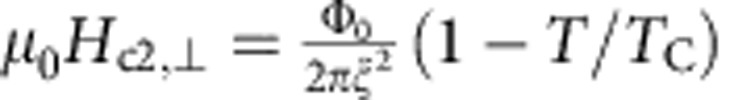
 and 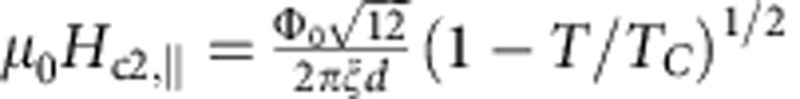, respectively^13^.

We applied also the Landau–Ginzburg analysis to a (001) LaAlO_3_/SrTiO_3_ sample using the same growth conditions as those used for the (110)-oriented samples. The analysis of the experimental data concludes that the coherence length is *ξ*≈40 nm and the superconducting thickness is *d*≈13 nm, in close agreement with the values previously reported^14^,^35^. We, thus, demonstrate in a quantitative manner that the spatial extension of superconductive (110) interfaces (*d*≈24–30 nm) is considerably larger than the one usually reported for (001) interfaces (*d*≈10–13 nm) (refs 14, 35).

The wider spatial extent of the (110)-2D state is also inferred from the analysis of the Pauli paramagnetic limit of the upper critical fields. For high-enough magnetic fields, the paramagnetic susceptibility induces a parallel alignment of the Cooper pair spins that eventually breaks them apart, giving a higher bound for the upper critical fields^18^,^36^. This value can be assessed as 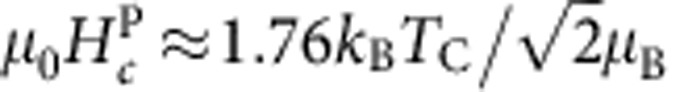, where *k*_B_ is the Boltzmann’s constant and *μ*_B_ is the Bohr magneton (assuming a *g* factor of 2)^18^,^36^. Although this upper bound is generally fulfilled, it is violated in some cases. One example is the case of ultrathin SrTiO_3_ 2D superconducting layers for which the values of *μ*_0_*H*_c2,‖_ were found to exceed largely the Pauli limit. This was explained by the large intrinsic spin–orbit coupling at interfaces, which becomes a prominent energy scale as the thickness is reduced^18^. The correlation between the spatial confinement and the anisotropy of the 2D superconductivity is also borne out in the (001) and (110) LaAlO_3_/SrTiO_3_ interfaces. Figure 2d summarizes this observation: the upper critical fields *μ*_0_*H*_c2,‖_ measured in (001) interfaces are significantly higher than those measured in (110) samples at any temperature. As a matter of fact, for the (001) interface the Pauli limit is already violated at temperatures below *T*≤220 mK, close to *T*_C_. Instead, the Pauli limit is only surpassed at temperatures *T*≤110 mK for the (110) interface, further away from the transition (Fig. 2d). Again, this is an indication of stronger 2DEG confinement at the (001) interface.

### Electrostatic modulation of 2D superconductivity

**Figure 3:**
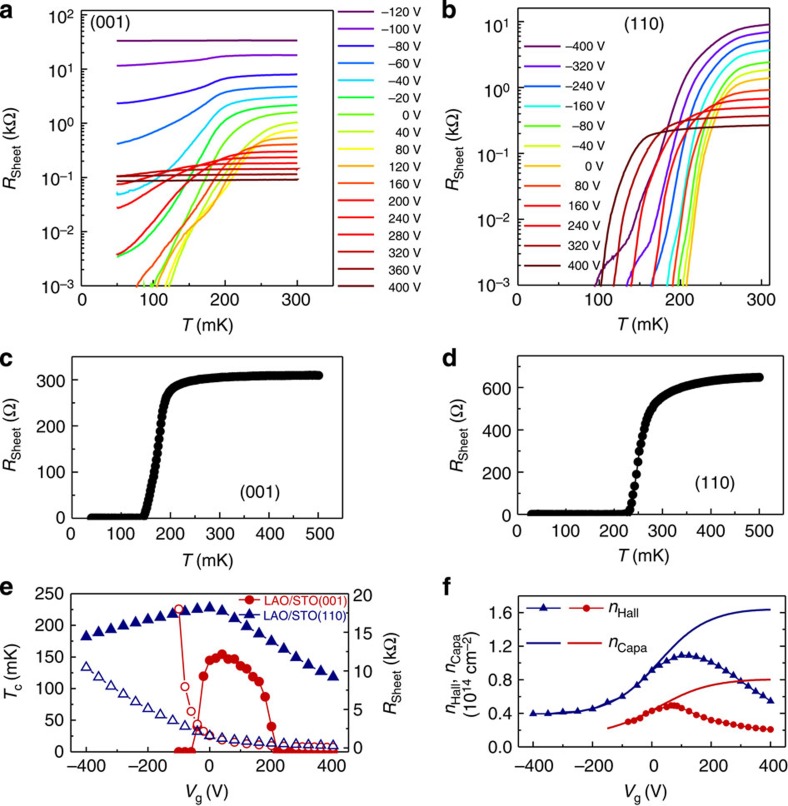
Electrostatic modulation of the 2D-superconductvity at the (001) and (110) interfaces. Temperature dependence of the resistance as a function of gate voltage for (**a**) (001)—and (**b**) (110)-oriented LaAlO_3_/SrTiO_3_ interfaces, respectively. In panels (**c**) and (**d**) we show the superconducting transitions in linear scale, for zero applied voltage, for (001) and (110) interfaces, respectively. (**e**) Superconducting *T*_C_ (filled symbols) and sheet resistance (open symbols) of (001)—and (110)-interfaces plotted against the gate voltage. (**f**) Hall carrier density of (001)- (circles) and (110)- (triangles) interfaces. Solid lines correspond to the carrier density obtained from the analysis of the capacitance.

The different 2DEG spatial extent has also consequences on the electrostatic modulation of the superconductivity. We performed electrostatic gating experiments in (001)- and (110)-oriented samples that were contacted by top and backgate electrodes and electric fields were applied in the range of *V*_g_=±400 V (Fig. 3). Positive/negative voltages correspond to the accumulation/depletion of electrons at the interface, respectively. Hall and capacitance experiments allowed us to obtain the sheet carrier density modulation as a function of the voltage *V*_g_ for both the film orientations. The curves of carrier density that we extract from Hall measurements exhibit a reduction of *n*_Hall_ for positive *V*_g_ (Fig. 3f). Such a feature is the hallmark of multiband conduction, in which high- and low- mobility carriers participate in the transport in the regime of accumulation, whereas only one type of carrier is relevant in the regime of depletion (*V*_g_≪0) (ref. 16). The total carrier density *n*_S_, comprising both heavy and light electron bands, can be obtained by experiments that measure the capacitance between the backgate and the 2DEG. In this case, the value of *n*_s_ is extracted by integration over the voltage range 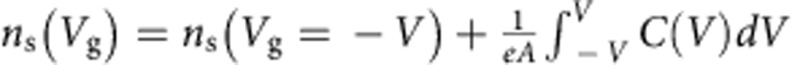, where *A* is the area of the capacitor. Note that, in agreement with the two-carrier scenario, only one band is involved in transport at negative *V*_g_ and *n*_S_ is superimposed to *n*_Hall_ within this range of applied voltages (Fig. 3f). Instead, in the regime of accumulation, *V*_g_>0, two bands are involved and *n*_S_ and *n*_Hall_ differ significantly^16^,^37^.

Figure 3e summarizes the results of the electrostatic gating experiments, where the superconducting transition temperature *T*_C_ and the resistance *R*_sheet_ at the normal state are plotted as a function of the gate voltage *V*_g_. We see that the carrier density is largely modulated for both orientations, with variations Δ*n*_s_=0.2−0.8 × 10^14^ cm^−2^ and Δ*n*_s_=0.4−1.6 × 10^14^ cm^−2^ for (001) and (110) interfaces, respectively. However, despite similar modulations of the carrier density for both orientations, their effects on the superconductivity are dramatically different depending on the crystal orientation. More specifically, the superconductivity of the (001)-interface could be suppressed for a range of applied fields (Fig. 3a), in agreement with previous reports^15^. At the (001) interface the *T*_C_(*V*_g_) curve exhibits a dome-like shape (Fig. 3e), indicating that superconductivity is suppressed at fields above *V*_g_≈+200 V and below *V*_g_≈−50 V. Instead, for (110) interfaces the superconducting state is never switched off by electric fields (Fig. 3b) and the transition temperature is modulated by at most about 50% (Fig. 3e). The much larger tunability of (001) interfaces with respect to (110) is again consistent with the narrower extension of 2DEGs at (001) wells.

### Modulation of the Rashba spin–orbit field

Previous works have demonstrated that there is a strong spin–orbit field that stems from a Rashba-type interaction at the LaAlO_3_/SrTiO_3_ interface^38^,^39^. As a result, an effective magnetic field *B*_SO_ is felt by electrons moving relativistically under the influence of the interface intensive electric fields *E*_0_=−∇*V*(*r*). Remarkably, the intensity of *B*_SO_ is directly related to electron hopping between *t*_2g_ orbitals that, although forbidden in the unperturbed system away from interfaces, are however allowed in the presence of the field *E*_0_ (ref. 40). In particular, *E*_0_ induces a polarization of the atomic orbitals, which break their symmetry and, as a consequence, allows a hybridization within the *t*_2g_ manifold in the metal–oxygen network that contributes to *B*_SO_ (refs 40, 41). Because of the different 2DEG band structure along (001) or (110), the spin–orbit field *B*_SO_ is expected to have a strong orientational dependence.

**Figure 4:**
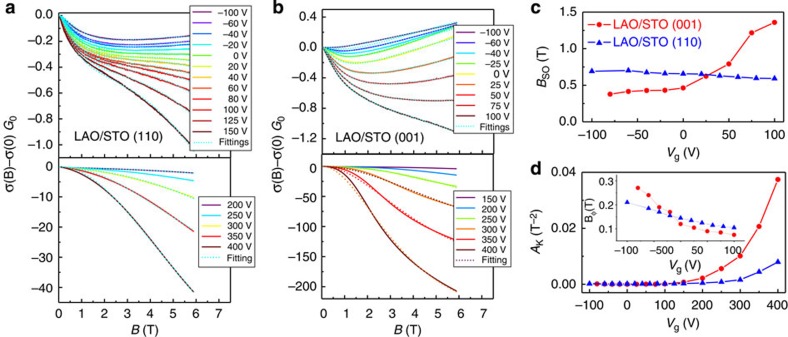
Analysis of the spin-orbit coupling at the (001) and (110) interfaces. The field-dependent magnetoconductance Δ*σ*(*B*) normalized by the quantum of conductance *G*_0_ was measured under different gate voltages *V*_g_. Data measured at *T*=3.3 K are shown for the (**a**) (110) and (**b**) (001) interfaces. Dashed lines are the fittings to equation (1) and the values of the applied voltage are indicated in the panels. The dependence of the spin–orbit term *B*_SO_ on the gate voltage *V*_g_ is plotted (**c**). The Kohler *A*_K_ and inelastic *B*_φ_ terms obtained from fittings to equation (1) are plotted in (**d**) and inset, respectively.

To probe the effects of orientational reconstruction on the spin–orbit term *B*_SO_, we analysed the field dependence of the magnetoconductance at the normal state recorded at a temperature *T*=3.3 K under applied electric fields (Fig. 4a,b). The experimental data were fitted to the expression^38^,^42^


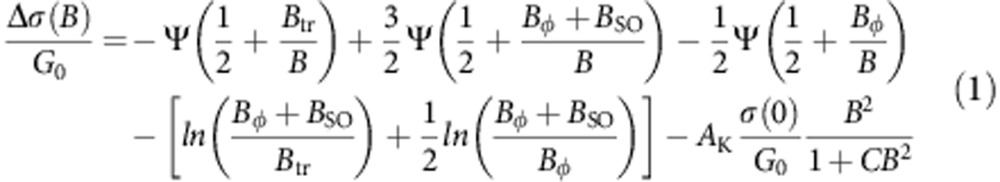


that describes the change of conductivity with field Δ*σ*(*B*) normalized by the quantum of conductance *G*_0_=*e*^2^/*πh* (refs 38, 42, 43). In equation (1), quantum corrections to the conductance in the 2D limit are described by the four first terms, where Ψ(*x*) is the digamma function, and *B*_tr_, *B*_φ_ and *B*_SO_ are the effective fields related to the elastic, inelastic and spin–orbit scattering terms, respectively^43^. Finally, the last term in equation 1, involving the parameters *A*_K_ and *C*, is the Kohler term that gives an account of orbital magnetoresistance. Fittings of the experimental data to equation (1) were excellent, as shown in Fig. 4a,b for both orientations and for different electric fields. The parameters 
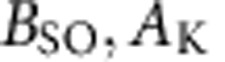
and *B*_φ_ extracted from these fittings are shown in Fig. 4c,d. It turns out that the Kohler term *A*_K_ became rather large at positive fields *V*_g_>+100 V (Fig. 4d), making difficult a precise evaluation of the spin–orbit term in the regime of strong electron accumulation. For that reason in Fig. 4c we plotted the evolution of the term *B*_SO_ restricted to the range *V*_g_=±100 V, where accurate values of the spin–orbit contribution can be obtained.

For the (001) sample, the values of *B*_SO_ that we obtained from fittings to equation (1) are in the same range as reported previously for the same orientation, with a similar asymmetric dependence of the spin–orbit field with *V*_g_ (refs 38, 43). In the regime of depletion (*V*_g_<0 V), the values of the spin–orbit field are *B*_SO_<0.5 T, whereas for electron accumulation (*V*_g_>0 V) the spin–orbit term rises up to *B*_SO_≈1.5 T. We thus observe a strong asymmetric field dependence of *B*_SO_ for the interfaces along (001). In contrast, the electrostatic modulation of *B*_SO_ is very weak along (110), and the spin–orbit field is largely unaffected by the electrostatic gating, with values restricted within a much narrower range *B*_SO_≈0.6–0.7 T (Fig. 4c). In brief, our analysis demonstrates that the Rashba spin–orbit fields at the (110) interfaces are substantially different from those along (001). This observation illustrates how band engineering based on crystal symmetry can be exploited to tailor the spin–dependent transport along SrTiO_3_-based quantum wells^44^,^45^.

## Discussion

**Figure 5:**
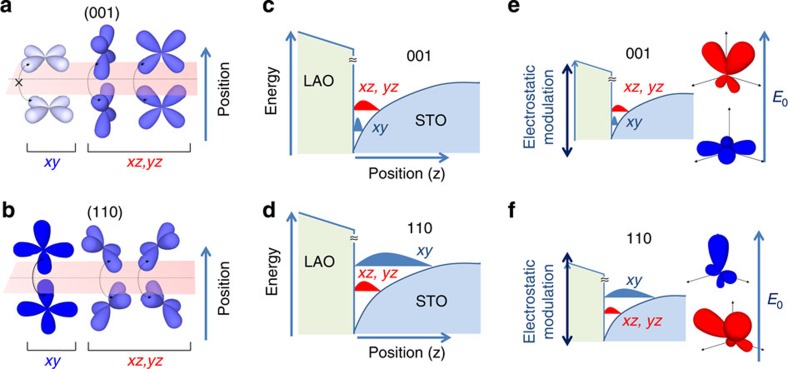
Energy landscape and orbital symmetries of 2DEGs at (001) and (110) LaAlO_3_/SrTiO_3_ interfaces. (**a**) Along (001), the overlapping of *d*_*xy*_ orbitals is very small, while it is moderately large for *d*_*xz*_/*d*_*yz*_ states. (**b**) Along (110), the overlapping of *d*_*xy*_ orbitals is the largest. Since the effective mass along the confinement direction is inversely proportional to the orbital overlapping, the hierarchy of masses is given by 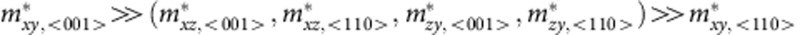. The rules of quantum physics in solids dictate the energy landscape in the quantum wells: (**c**) Orbitals with symmetry *d*_*xy*_ lie at the bottom of (001) quantum wells, while *d*_*xz*_/*d*_*yz*_ are higher in energy; (**d**) Along (110), the *d*_*xz*_/*d*_*yz*_ states have lower energy and the *d*_*xy*_ levels are at the top. The different energy hierarchy of orbitals determines a larger spatial extension for 2DEGs along (110) as compared with (001). (**e**) Sketches how the *d*_*xy*_ and *d*_*xz*_/*d*_*yz*_ orbitals are distorted by the inversion symmetry breaking field *E*_0_ at the interface. Along (001), *d*_*xy*_ states have a small projection along the out-of-plane direction and they are weakly polarized by *E*_0_, whereas *d*_*xz*_/*d*_*yz*_ states project along the direction of confinement and are largely polarized. (**f**) Instead, along (110), both kinds of orbitals are affected similarly by the interface fields. Within the range of applied fields, the electrostatic modulation has dissimilar effects on the spin–orbit fields *B*_SO_. Along (001), only *d*_*xy*_ orbitals are filled at *V*_g_<0, whereas at *V*_g_>0 the *d*_*xz*_/*d*_*yz*_ states are progressively occupied. Thus, at low electrostatic doping, *B*_SO_ is smaller because of the relatively small atomic orbital polarization of *d*_*xy*_ states, whereas the population of *d*_*xz*_/*d*_*yz*_ orbitals at positive *V*_g_ increases significantly *B*_SO_. Instead, along (110) the dependence of *B*_SO_ on the applied fields is weak, because all *t*_2g_ orbitals are expected to undergo polarizations of similar strength.

The different spatial extension of the quantum wells along (001) and (110) and the different behaviour of the Rashba spin–orbit fields can be elucidated on the grounds of the modulation of the 2DEG sub-band structure observed in the experiments^24^ that, in turn, can be understood using the fundamental concepts of quantum physics of solids. When we consider the orbitals of *t*_2g_ electrons that are confined along (001) or (110), the quantum well entrapment of *d*_xy_, *d*_xz_ and *d*_yz_ wavefunctions produces an energy splitting between the different eigenstates that is inversely proportional to their effective masses along the confinement direction^46^. Figure 5 illustrates schematically the arguments that we expose in the following. Note that although the full complexity of the quantum sub-band structure^47^,^48^ is ignored in this Figure—as we depict only one sub-band for each type of orbital— the essential physics is captured. More specifically, for confinement along (001), *π*-type bonding between *d*_xy_ states leads to small wavefunction overlapping and large effective mass, while along (110) *σ*-like bonds between *d*_xy_ orbitals lead to much smaller effective mass (Fig. 5a,b). Instead, the overlapping of *d*_xz_/*d*_yz_ states has intermediate values for both the orientations. This results in a hierarchy of out-of-plane effective masses given by 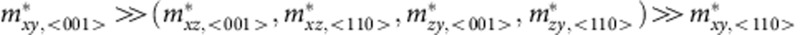 that, in turn, yields the energy orbital landscape outlined in Fig. 5c,d, which is in agreement with the 2DEG sub-band hierarchy observed in X-ray linear dichroism experiments^46^,^49^.

As a consequence of the observed rearrangement of orbital symmetries, the spatial extension of the 2DEG must change significantly with the crystal orientation. In this respect, Fig. 5c,d plot schematically the carrier spatial distributions of *d*_*xy*_*, d*_*xz*_ and *d*_*yz*_ states: along (001) the first *d*_xy_ sub-band is expected to be at the bottom of the well, with little spatial spread; on the contrary, along (110) the *d*_*xy*_ level raises its energy above the *d*_*xz*_*/d*_*yz*_ states, and its spatial extent is considerably larger. In addition to orbital occupancy, contributions from the anisotropic character of the dielectric constant tensor may also influence the 2DEG spatial extent. Therefore, the modulation of the orbital hierarchy described here provides a natural explanation for the distinct anisotropy of the 2D superconductivity and spatial extension for quantum wells oriented along (001) and (110).

By the same token, the redistribution of the orbital sub-band hierarchy also explains the distinctive dependence of the Rashba spin–orbit fields on the orientation. As mentioned above, the interface electric field *E*_0_ induces a polarization of the atomic orbitals that breaks their symmetry along the direction of the quantum well. This enables new covalent channels within the *t*_2g_ manifold and the oxygen network that contributes to *B*_SO_ (refs 40, 41). The key point is to recognize that orbitals with large projections over the normal to the interface are those more sensitive to the inversion symmetry breaking fields *E*_0_, giving larger Rashba effects^40^,^41^. Such atomic orbital polarization is graphically depicted in Fig. 5e in the form of spatially distorted orbitals.

In the light of these observations, the asymmetric modulation of *B*_SO_ with field along (001), Fig. 4c, can be explained because *d*_xy_ orbitals are weakly polarized due to their minimal projection along the confinement direction, while *d*_*xz*_/*d*_*yz*_ states have much stronger spatial asymmetry (Fig. 5e). As a result of the 2DEG sub-band hierarchy along (001), the electrostatic modulation of orbital occupancy is anticipated to give a significant variation of *B*_SO_ as a function of the orbital occupancy: at *V*_g_<0 V only *d*_xy_ orbitals are populated; the orbital polarization is weak (Fig. 5e) and *B*_SO_ is relatively small. In contrast, as we enter the regime of accumulation and *d*_*xz*_*/d*_*yz*_ bands start to be filled, the spin–orbit term begins to increase significantly, in agreement with the significantly larger orbital polarization of these orbitals.

The situation is radically different for the (110) interface. Now, the electrostatic modulation of *B*_SO_ is very weak and the spin–orbit field is largely unaffected by the electrostatic gating. This behaviour, which may seem surprising in the light of the modulation of carrier density with electrostatic gating (Fig. 4d), can be well understood on the grounds of the similar atomic orbital polarizations of *d*_*xy*_ and *d*_*xz*_*/d*_*yz*_ orbitals along (110), Fig. 5e. Indeed, along (110), all *t*_2g_ orbitals are expected to undergo similarly strong polarizations (Fig. 5e) and, therefore, the spin–orbit field *B*_SO_ is expected to have a rather weak dependence on the applied field, as confirmed by the experiments.

In summary, we have shown that the orbital reconstruction that occurs for LaAlO_3_/SrTiO_3_ quantum wells confined along two different directions, (001) and (110) has a deep impact on the physical properties of these 2DEGs. We claim that the different energy landscapes and hierarchy of orbital symmetries are behind the observed differences in the 2DEG spatial extensions and spin–orbit fields. The analysis of the 2D superconductivity is consistent with 2DEGs extending spatially over (110) at larger distances than at (001) interfaces. At the same time, electrostatic gating experiments have provided relevant clues to understand the distinctive spatial distribution of *t*_2g_ states with respect to the interface that results from the modified energy sub-band hierarchy and the renormalization of the associated effective band masses. Our work shows that crystal symmetry is an extra degree of freedom to realize different 2DEG band reconstructions at the LaAlO_3_/SrTiO_3_ interface, thus allowing a selective occupancy of states of different symmetry. Such new perspective for 2DEG band engineering is very alluring, as it opens new research fields to extend our current understanding of the link between orbital symmetry and magnetism and superconductivity at LaAlO_3_/SrTiO_3_ quantum wells.

## Methods

### Sample preparation

For the growth of (110)-oriented samples, the SrTiO_3_ substrates were treated in a dedicated furnace at 1,100 °C for 2 h under ambient conditions^26^,^27^. Samples with (001) orientation were grown on TiO_2_-terminated SrTiO_3_ substrates. The TiO_2_ termination of the SrTiO_3_(001) single crystals was obtained by chemical treatment followed by thermal annealing^50^,^51^. LaAlO_3_ thin films were grown by pulsed laser deposition (*λ*=248 nm) monitored by high pressure reflection high-energy electron diffraction. The substrates were heated from room temperature to deposition temperature (850 °C) in an oxygen partial pressure *P*_O2_=0.1 mbar. During deposition, the LaAlO_3_ was grown under a pressure *P*_O2_=10^−4^ mbar and 1-Hz repetition rate, with laser pulse energy of around 26 mJ. Films with thickness 7, 8, 10 and 14 MLs were prepared on (110) substrates, whereas the (001)-oriented sample had a LaAlO_3_ thickness of 10 MLs. At the end of the deposition, samples were cooled down in an oxygen rich atmosphere to minimize the formation of oxygen vacancies that could lead to extrinsic mechanisms of conduction. More specifically, the samples were cooled from *T*=850 to 750 °C under a pressure *P*_O2_=0.3 mbar and under *P*_O2_=200 mbar from *T*=750 °C down to room temperature, including a dwell time of 1 h at 600 °C.

### Magnetotransport

The electrical characterization was performed by using six-contact arrangement in Hall geometry, from which the sheet resistance, sheet carrier density and electron mobility were extracted as a function of temperature and gate voltage. The current was injected along the in-plane (001) direction in (110)-interfaces. The LaAlO_3_/SrTiO_3_ interface was contacted via ultrasonic wire bonder with Al wires. Measurements at temperatures below 1.8 K were measured in a dilution cryostat by applying 50 nA AC current of frequency 13.67 Hz. For the estimation of critical field, the magnetic field was applied parallel and perpendicular to the sample plane with sweep rates of 1.6 mT s^−1^. For the measurement of the parallel critical magnetic field of the 110 samples, the field was applied in the same direction than the current, that is, along the in-plane (001). Electric fields were applied using voltage source. No leakage current (<5 nA) was detected up to largest applied voltages ±400 V.

### Transmission electron microscopy

STEM-HAADF images were acquired with a NION UltraSTEM, equipped with a 5th order NION aberration corrector and operated at 200 kV, and in a FEI Titan (60–300 kV) STEM operated at 300 kV, equipped with a probe Cs corrector from CEOS, a monochromator and a high-brightness field-emission gun (X-FEG). HAADF signals for the samples were collected from the detector inner-angles of ~86 and ~60 mrad for the NION and FEI Titan microscopes, respectively. Specimens for STEM were prepared by conventional methods, by grinding, dimpling and argon ion milling.

## Author contributions

G.H. designed and conceived the experiments with help from J.F., N.B. and J.L. Low-temperature magnetotransport experiments were done by G.S., A.J. and N.B. The analysis of low-temperature magnetotransport data was done by G.S., N.B. and G.H. The thin film preparation, *in situ* reflection high-energy electron diffraction characterization and structural and surface morphology analysis were done by M.S., N.D. and F.S. J.G., and M.V. made all the characterization and analysis of structural characterization by STEM-HAADF. G.H. wrote the paper. All authors discussed the data and commented on the paper.

## Additional information

**How to cite this article**: Herranz, G. *et al.* Engineering two-dimensional superconductivity and Rashba spin–orbit coupling in LaAlO_3_/SrTiO_3_ quantum wells by selective orbital occupancy. *Nat. Commun.* 6:6028 doi: 10.1038/ncomms7028 (2015).

## Supplementary Material

Supplementary Information Supplementary Figures 1-3 and Supplementary References
