# Supplementary material for: Engineering two-dimensional superconductivity and Rashba spin–orbit coupling in LaAlO3/SrTiO3 quantum wells by selective orbital occupancy
Source: Nat Commun. 2015 Jan 13;6:6028. doi: 10.1038/ncomms7028 (PMC4308716; doi:10.1038/ncomms7028)
Supplement: Supplementary Information — Supplementary Figures 1-3 and Supplementary References [file ncomms7028-s1.pdf]

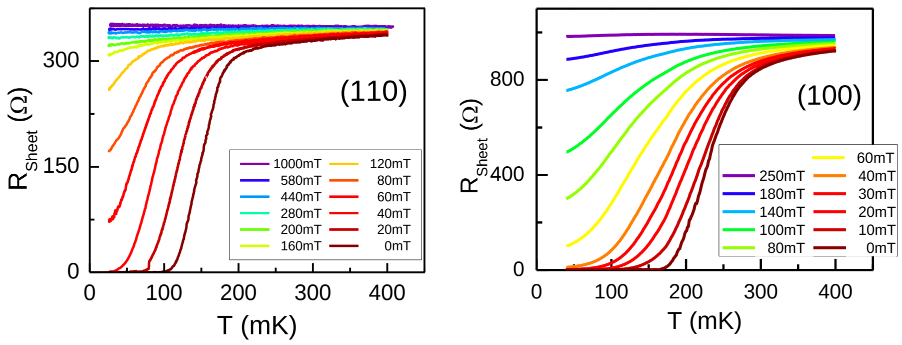

Supplementary Figure 1| Out-of-plane upper critical fields at (001) and (110) $LaAlO_3/SrTiO_3$ interfaces.

Sheet resistance of (a) the (110)-interface with *t* = 14 MLs and (b) the (001)-interface with *t* = 10 MLs, under magnetic fields applied normal to the interface. The field values are indicated in the panels of Supplementary Figure 1.

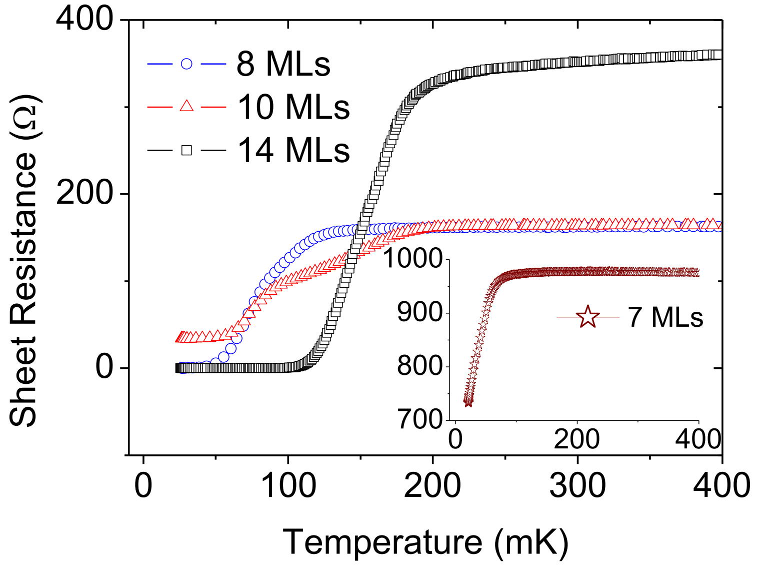

Supplementary Figure 2| Superconductive transitions at the (110) $LaAlO_3/SrTiO_3$ interface.

We defined the onset of the transition as the temperature where the resistance falls to 90% of its normal state value (measured at T = 400 mK). We observed that the superconductive transition temperature T_C of (110)-oriented interfaces was strongly dependent on the sample, being the highest for t = 14 MLs (T_C ≈ 195 mK), whereas lower values were observed for t = 10 MLs (T_C ≈ 166.5 mK), t = 8 MLs (T_C ≈ 115 mK) and t = 7 MLs (T_C ≈ 45 mK) (Supplementary Figure 1). Samples with t = 14 MLs and t = 8 MLs exhibited the narrowest transition widths ΔT_C ≈ 40 mK (defined between 20% and 80% of the normal state resistance), comparable to the ΔT_C observed in superconductive (001)-$LaAlO_3/SrTiO_3$ as well as (001)-$LaTiO_3/SrTiO_3$ [1-3] interfaces The other samples exhibited significantly broader transitions, indicating more inhomogeneous electronic states. This observation is more perceptible in sample t = 10 MLs that exhibits a complex transition to superconductivity with two kinks.

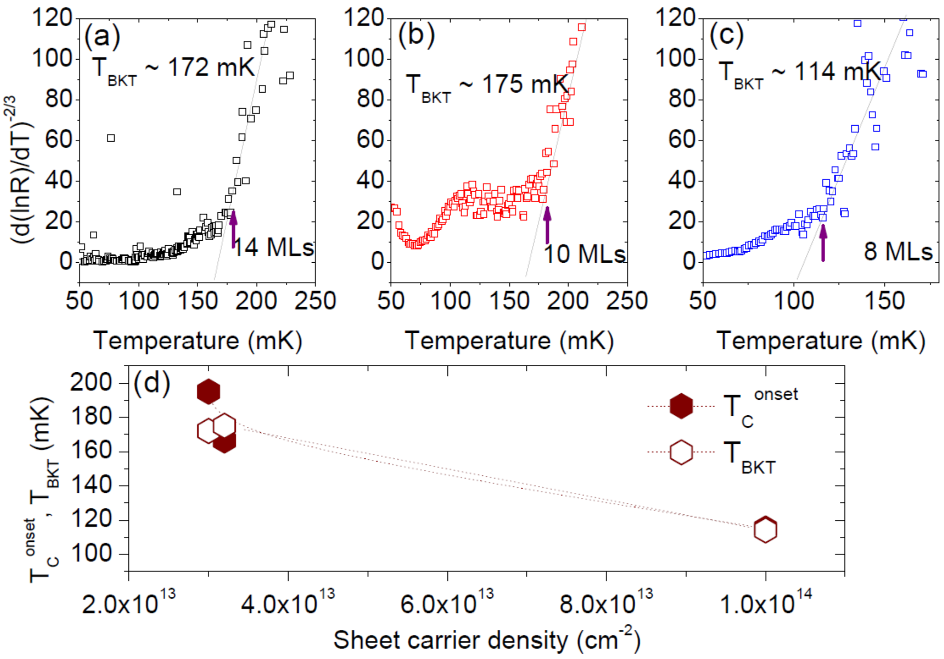

Supplementary Figure 3| Analysis of the 2D-superconductivity transition: the Berezinskii-Kosterlitz-Thouless (BKT) model.

In the 2D limit, it is well established that superconductivity is suppressed by vortex-antivortex pair unbinding by thermal fluctuations, so that the transition to the 2D superconductive state belongs to the Berezinskii-Kosterlitz-Thouless (BKT) universality class. In this scenario, above the transition temperature T_{BKT} the free motion of vortices triggers the transition from the 2D superconductive towards the normal state. Within a narrow region around T_{BKT} the BKT theory predicts specific laws for the temperature dependence of the resistance R, giving a quantifiable criterion to assess the dimensional character of the superconductive state. In particular, it is expected that $\left[\partial lnR/_{\partial T}\right]^{-2/_3}$ should scale linearly with $(T - T_{BKT})$ for a certain region around T_{BKT} [4][As seen in panels a-c, similarly to (001)- LAO/STO interfaces, the $\left[\partial lnR/_{\partial T}\right]^{-2/_3}$ data corresponding to (110) interfaces fall approximately on a straight line above a certain temperature that –following the same protocol as described in the analogous analysis of (001)-interfaces[1] – we identify as the T_{BKT} transition temperature. Therefore, the temperature T_{BKT} is at the boundary between the two regimes (superconductive/normal) giving rise to a prominent kink in the $\left[\partial lnR/_{\partial T}\right]^{2/_3}$ plot. We note that the values of T_{BKT} thus obtained are in the same range as those found in (001)-$LaAlO_3$/$SrTiO_3$. We find that the T_{BKT} values correlate with T_C (panel d), both being strongly dependent on the sheet carrier density n_{sheet} in the normal state, a feature that is reminiscent of the strong dependence of the bulk superconductive transition on carrier density.

Supplementary References

1. Reyren, N. *et al.*, Superconducting Interfaces Between Insulating Oxides. *Science* **317**, 1196-1199 (2007).

2. Biscaras, J. *et al.* Two-dimensional superconductivity at a Mott insulator/band insulator interface $LaTiO_3/SrTiO_3$. *Nat. Commun.* **1**, 89 (2010).

3. Biscaras, J. *et al.*, Two-Dimensional Superconducting Phase in $LaTiO_3/SrTiO_3$ Heterostructures Induced by High-Mobility Carrier Doping. *Phys. Rev. Lett.* **108**, 247004 (2012).

4 She, J.-H. & Balatsky, A.V. Berezinskii-Kosterlitz-Thouless Transition to the Superconducting State of Heavy-Fermion Superlattices. *Phys. Rev. Lett.* **109**, 077002 (2012).
